# Gel Immersion Endoscopic Mucosal Resection With Submucosal Injection for Superficial Esophageal Squamous Neoplasms (With Video)

**DOI:** 10.1002/deo2.70444

**Published:** 2026-10-07

**Authors:** Takahiro Uda, Yuji Urabe, Tomoyuki Gurita, Yoshiki Hatsushika, Satoshi Masuda, Yukiko Sako, Takeo Nakamura, Yuki Kamigaichi, Takeshi Takasago, Hidenori Tanaka, Akiyoshi Tsuboi, Ken Yamashita, Yuichi Hiyama, Yoshihiro Kishida, Hidehiko Takigawa, Akira Ishikawa, Toshio Kuwai, Shinji Mii, Shiro Oka

**Affiliations:** ^1^ Department of Gastroenterology Graduate School of Biomedical and Health Sciences Hiroshima University Hiroshima Japan; ^2^ Department of Clinical Research Center Hiroshima University Hospital Hiroshima Japan; ^3^ Department of Molecular Pathology Graduate School of Biomedical and Health Sciences Hiroshima University Hiroshima Japan; ^4^ Department of Gastrointestinal Endoscopy and Medicine Hiroshima University Hospital Hiroshima Japan

**Keywords:** endoscopic mucosal resection, endoscopic resection, esophageal squamous neoplasms, gel immersion, minimally invasive treatment

## Abstract

**Objectives:**

Gel immersion can provide a stable immersion environment during endoscopic mucosal resection (EMR). However, its application in superficial esophageal squamous lesions remains limited. In this study, we evaluated a gel immersion EMR (GI‐EMR) technique in which submucosal injection was intentionally performed before snaring for selected small superficial esophageal squamous lesions.

**Methods:**

This single‐center, retrospective case series included consecutive patients who underwent GI‐EMR with submucosal injection for superficial esophageal squamous lesions ≤15 mm in size between January and March 2026. The primary outcome was the histological en bloc resection rate, while secondary outcomes included technical success, endoscopic en bloc resection rate, procedure time, adverse events, and histopathological findings.

**Results:**

GI‐EMR with submucosal injection was attempted for 13 lesions, achieving a technical success rate of 92% (12/13). One procedure was discontinued because adequate lifting could not be achieved due to post‐chemoradiotherapy scarring. The endoscopic and histological en bloc resection rates were both 85% (11/13) on an intention‐to‐treat basis. The median procedure time was 4.25 min (range, 2.0–11.0 min). No intraoperative or delayed perforation, post‐procedure bleeding, or gel‐related adverse events occurred.

**Conclusions:**

GI‐EMR with submucosal injection was technically feasible for selected superficial esophageal squamous lesions ≤15 mm, with no serious procedure‐related adverse events observed in this small case series.

**Trial Registration:**

N/A

## 1 Introduction

Endoscopic resection is commonly performed to treat superficial esophageal cancer in Japan, with endoscopic submucosal dissection (ESD) serving as the standard approach [1]. ESD achieves high en bloc and curative resection rates with a low risk of local recurrence. However, esophageal ESD is technically demanding and requires a prolonged procedure time [2]. Furthermore, extended upper gastrointestinal ESD is associated with an increased risk of postoperative pneumonia, underscoring the importance of minimizing sedation‐related respiratory adverse events [3].

Endoscopic mucosal resection (EMR) is a simpler and more rapid alternative; however, it is associated with a risk of piecemeal resection, which may compromise pathological margin assessment and increase the risk of residual disease and local recurrence [4]. Several EMR techniques, including cap‐assisted EMR (EMR‐C) [5, 6] and endoscopic variceal ligation–based resection [7], have been developed for the endoscopic resection of esophageal lesions. However, these techniques often require dedicated devices and may limit direct visualization of tumor margins during snaring.

Gel immersion (GI) endoscopy is a technique in which a viscous gel is injected into the gastrointestinal lumen instead of air or water to maintain a clear and stable visual field during endoscopic procedures. The viscosity of the gel helps suppress the dispersion of blood and mucus and may improve lesion visibility and tissue manipulation during resection. This technique has recently emerged as a novel approach to improve visualization and procedural stability, with growing evidence supporting its use in the duodenum, stomach, and colon [8, 9, 10, 11]. Nakano et al. [12] reported that GI led to improved visualization and reduced sedative dosage during the ESD of esophageal lesions, particularly for gravity‐dependent lesions. However, its application in superficial esophageal neoplasms remains limited.

In the esophagus, stable visualization during snaring can be challenging due to the narrow lumen and presence of mucus or bleeding. Under such conditions, GI may provide a more stable visual field than air‐ or water‐based techniques, facilitating lesion capture without the need for dedicated resection devices. These features indicate that GI‐EMR may enable rapid, visually controlled resection in selected cases.

In this study, we evaluated a GI‐EMR technique specifically adapted for the esophagus, in which submucosal injection was intentionally performed before snaring. We aimed to assess the feasibility and safety of this technique for the rapid, visually controlled resection of selected small superficial esophageal squamous lesions.

## 2 Patients and Methods

### 2.1 Study Design and Patients

A single‐center, retrospective case series was conducted at Hiroshima University Hospital. Consecutive patients who underwent attempted GI‐EMR with submucosal injection for superficial esophageal squamous lesions between January and March 2026 were included.

GI‐EMR with submucosal injection had not been performed at our institution before January 2026. The technique was first introduced into routine clinical practice in January 2026 for a superficial esophageal lesion, which was reported in a prior case report [13]. The present study retrospectively evaluated consecutive patients who underwent attempted GI‐EMR with submucosal injection during the initial period of its clinical use between January and March 2026, including that initial case. During this period, the technique was performed, in principle, for all consecutive superficial esophageal squamous lesions ≤15 mm that were endoscopically diagnosed as mucosal lesions and considered amenable to snare resection. Lesions larger than 15 mm or those considered unsuitable for snare‐based resection, including lesions with suspected deeper invasion, were generally treated with ESD or other appropriate treatment modalities. Conventional EMR was not routinely selected for lesions meeting the above criteria during this period. The technique continued to be used in routine clinical practice after the study period.

Given that this study focused on short‐term technical outcomes, resection outcomes, and safety, no patient‐level exclusion criteria were applied, including age‐based criteria. The presence of multiple esophageal lesions, synchronous or metachronous cancers, or a history of prior treatment for esophageal cancer was not considered an exclusion criterion.

The lesion‐level inclusion criterion was a superficial esophageal squamous lesion measuring ≤15 mm and diagnosed preoperatively as a mucosal lesion by endoscopy. No restriction was applied based on the longitudinal or circumferential location of the lesion within the esophagus.

This study was approved by the Institutional Review Board of Hiroshima University Hospital (approval number: E2025‐0296) and was conducted in accordance with the principles of the Declaration of Helsinki. Informed consent was obtained using an opt‐out approach through in‐hospital notification.

### 2.2 Rationale for Lesion Selection

Eligible lesions were defined as superficial esophageal squamous lesions measuring ≤15 mm that had been diagnosed preoperatively as mucosal cancers confined to the epithelium (EP) or lamina propria mucosae (LPM). Only lesions ≤15 mm were selected because previous studies have shown that EMR‐based techniques can achieve favorable en bloc resection outcomes for lesions of this size [14, 15].

### 2.3 Endoscopic Procedure: GI‐EMR With Submucosal Injection

All procedures were performed by endoscopists experienced in esophageal endoscopic therapy. Procedures were generally performed under intravenous sedation with midazolam and haloperidol. General anesthesia with endotracheal intubation was used in one patient with a cervical esophageal lesion, considering the location. All procedures were performed with the patient in the left lateral position. Among the nine patients who underwent the procedure under intravenous sedation, an overtube was used in seven patients. Overtube use was not standardized and was determined at the discretion of the endoscopist based on procedural considerations, including endoscopic maneuverability. In the patient who underwent general anesthesia with endotracheal intubation, an overtube was not used. A high‐definition endoscope (GIF‐H290T; Olympus, Tokyo, Japan, or EG‐860TP; Fujifilm, Tokyo, Japan) was used, without a distal attachment in any case.

The lesion extent was initially assessed using a combination of white‐light imaging, narrow‐band imaging, and iodine staining. GI‐EMR with submucosal injection was conducted according to the following procedure (Figure 1; Video S1):

1. Marking around the lesion was performed.
2. Any air and mucus in the esophagus were thoroughly aspirated to collapse the lumen.
3. Approximately 50 mL of VISCOCLEAR (Otsuka Pharmaceutical Factory, Tokushima, Japan) was initially injected manually using a syringe through the accessory channel to achieve complete immersion of the lesion. Additional gel was administered manually using a syringe as needed to maintain adequate immersion and visualization, with the total amount generally not exceeding 100 mL per procedure.
4. After GI, a hyaluronic acid solution (MucoUp; Boston Scientific, Tokyo, Japan) was injected into the submucosal layer beneath the lesion to create a submucosal cushion.
5. The lesion was captured using a 15‐mm snare (SnareMaster; Olympus) and resected by EMR.
6. The resected specimen was retrieved and subjected to histopathological evaluation for resection margins and en bloc resection.

**FIGURE 1:**
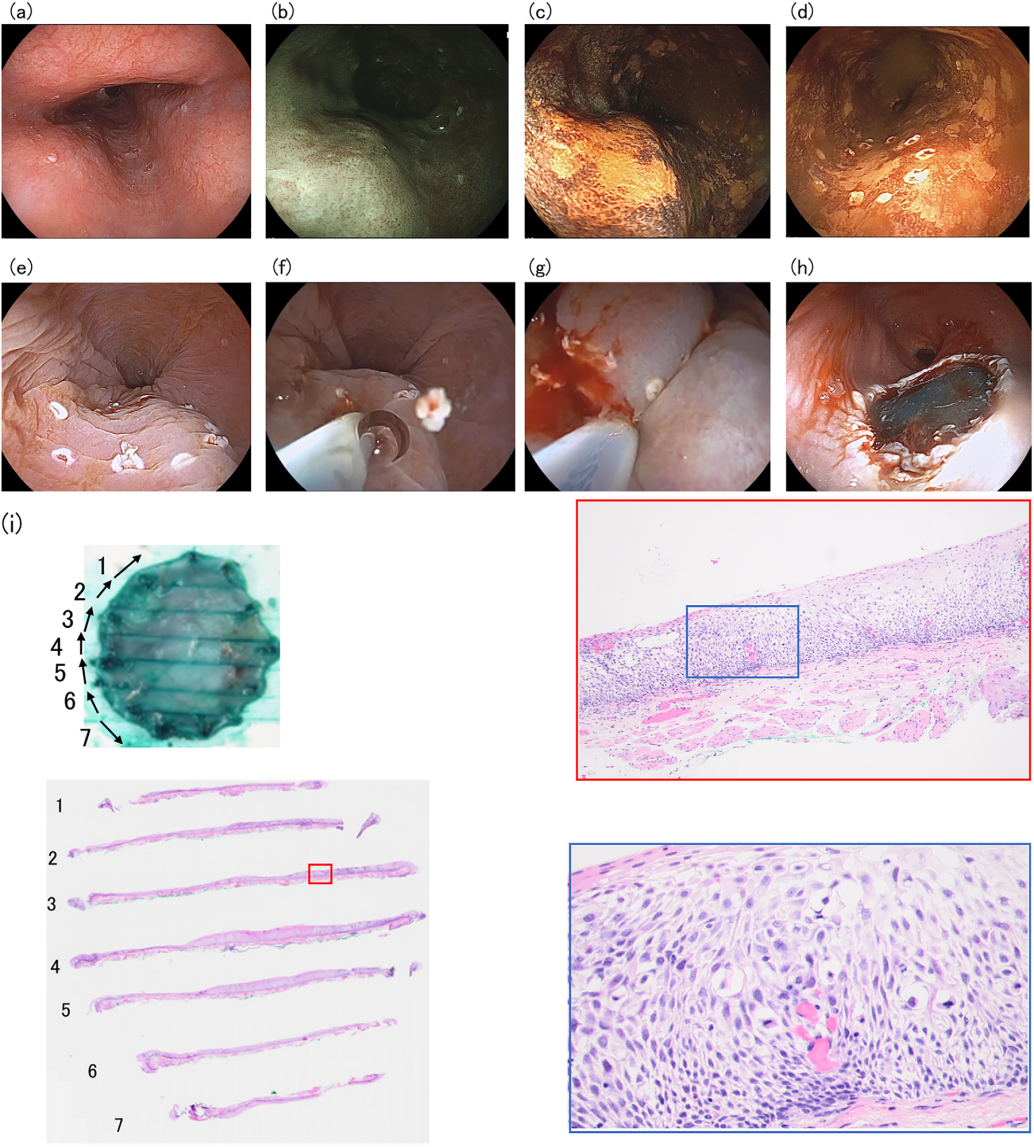
Overview of a representative case of gel immersion endoscopic mucosal resection with submucosal injection for a superficial esophageal squamous lesion, including histopathological findings. (a) White‐light imaging showing a reddish depressed lesion. (b) Narrow‐band imaging demonstrating a brownish area. (c) Iodine staining showing an unstained area corresponding to the lesion. (d) Marking performed around the lesion following iodine staining. (e) Gel immersion of the lesion. (f) Submucosal injection beneath the lesion. (g) Snaring under gel immersion. (h) The post‐resection ulcer base. (i) Histopathological examination of the resected specimen confirming squamous cell carcinoma confined to the epithelium (EP).

If required, hemostasis was achieved using a Coagrasper (Olympus). An electrosurgical unit (VIO3; ERBE Elektromedizin GmbH, Tübingen, Germany) was used for all procedures. Marking was conducted using soft coagulation (effect 6.0). Resection was performed using the Endo Cut I mode (effect 1, duration 3, interval 1), and hemostasis was achieved using soft coagulation (effect 6.0).

Following the procedure, all patients were hospitalized for observation. Oral intake of water was initiated on the day after the procedure, with a soft diet initiated on postoperative day 2. Patients were discharged on the following day, after confirming an uneventful clinical course.

### 2.4 Outcome Measures

The primary outcome was the histological en bloc resection rate. Histological en bloc resection was defined as resection of the lesion in a single piece with histologically negative horizontal and vertical margins. The secondary outcomes included the technical success rate, endoscopic en bloc resection rate, procedure time, adverse events, and histopathological findings. Technical success was defined as completion of the planned GI‐EMR with submucosal injection procedure and was calculated per attempted lesion, including cases in which resection could not be completed. Endoscopic en bloc resection was defined as resection of the lesion in a single piece, irrespective of the histological margin status. Endoscopic and histological en bloc resection rates were calculated per attempted lesion. Lesions in which the procedure could not be completed were considered failures for these outcomes. The procedure time was defined as the interval from the start of submucosal injection to the completion of resection.

Adverse events included intraoperative perforation, delayed perforation, post‐procedure bleeding, and gel‐related adverse events. Post‐procedure bleeding was defined as bleeding requiring any additional intervention, such as endoscopic hemostasis or blood transfusion. Perforation was defined as a full‐thickness muscular layer defect identified endoscopically or free air on imaging. The maximum thickness of the submucosal layer in the resected specimen was evaluated histologically as the greatest distance from the lower border of the muscularis mucosae to the deepest resection margin.

### 2.5 Statistical Analysis

Continuous variables are presented as median values with ranges, and categorical variables are presented as frequencies and percentages. No comparative statistical analyses were performed because this was a descriptive case series. All statistical summaries were calculated using JMP 18 software (JMP Statistical Discovery, Cary, NC, USA).

## 3 Results

### 3.1 Patients and Lesion Characteristics

The detailed characteristics of all included lesions are shown in Table 1, while the aggregated patient and lesion characteristics are presented in Table 2. Overall, 10 consecutive patients with 13 lesions were included. The median patient age was 67 years (range, 56–77 years), and one patient (10%) was receiving antithrombotic therapy.

**TABLE 1:** Detailed characteristics and treatment outcomes of all lesions treated with gel immersion endoscopic mucosal resection (GI‐EMR) with submucosal injection.

Case	Lesion	Age, years	Sex	Tumor location	Lesion background	Macroscopic type	Circumferential extension	Endoscopic tumor size, mm	Resected specimen size, mm	Histopathological diagnosis	Endoscopic en bloc resection	Histological en bloc resection	Procedure time, min
1	1	56	F	Middle	Non‐scarred	0‐IIb	<1/4	2	4 × 3	IN	Achieved	Achieved	3.0
1	2			Middle	Non‐scarred	0‐IIb	<1/4	2	3 × 3	IN	Achieved	Achieved	2.5
1	3			Middle	Non‐scarred	0‐IIb	<1/4	2	6 × 6	IN	Achieved	Achieved	2.0
2	4	71	M	Middle	Non‐scarred	0‐IIc	<1/4	10	10 × 8	EP‐Ca	Achieved	Achieved	4.5
3	5	69	M	Lower	Non‐scarred	0‐IIc	<1/4	8	8 × 8	IN	Achieved	Achieved	5.5
4	6	75	M	Middle	Non‐scarred	0‐IIb	<1/4	5	15 × 10	EP‐Ca	Achieved	Achieved	4.0
5	7	65	M	Upper	Non‐scarred	0‐IIc	<1/4	5	10 × 10	EP‐Ca	Achieved	Achieved	4.0
6	8	72	M	Upper	Non‐scarred	0‐IIc	<1/4	15	15 × 12	LPM‐Ca	Achieved	Achieved	8.0
7	9	65	M	Upper	Non‐scarred	0‐IIc	<1/4	5	9 × 9	EP‐Ca	Achieved	Achieved	5.0
7	10			Upper	Non‐scarred	0‐IIc	<1/4	2	9 × 9	EP‐Ca	Achieved	Achieved	2.0
8	11	77	M	Middle	Non‐scarred	0‐IIc	<1/4	10	15 × 15	EP‐Ca	Achieved	Achieved	6.0
9	12	64	M	Middle	Post‐ESD scar	0‐IIc	<1/4	10	20 × 15	IN	Not achieved	Not achieved (Rx)	11.0
10	13	63	M	Middle	Post‐CRT scar	0‐IIc	<1/4	5	NA	NA	Not completed	NA	NA

Abbreviations: CRT, chemoradiotherapy; EP‐Ca, epithelial carcinoma; ESD, endoscopic submucosal dissection; IN, intraepithelial neoplasia; LPM‐Ca, lamina propria mucosae carcinoma. NA, not applicable.

Age and sex were analyzed per patient (*n* = 10), whereas the remaining variables were analyzed per lesion (*n* = 13).

**TABLE 2:** Overview of aggregated patient and lesion characteristics.

Variables	*n* = 10 patients/13 lesions
**Patients**	
Age, median (range), years	67 (56–77)
Sex, male/female	9/1
Antithrombotic therapy, *n* (%)	1 (10%)
**Lesions**	
Location, *n* (%)	
Upper esophagus (Ut)	4 (31%)
Middle esophagus (Mt)	8 (61%)
Lower esophagus (Lt)	1 (8%)
Circumferential extension, *n* (%)	
<1/4	13 (100%)
1/4–1/2	0 (0%)
>1/2	0 (0%)
Macroscopic type, *n* (%)	
0‐IIb	3 (23%)
0‐IIc	10 (77%)
Endoscopic tumor size, median (range), mm	5 (2–15)

Age, sex, and antithrombotic therapy were analyzed per patient (*n* = 10), whereas lesion‐related variables were analyzed per lesion (*n* = 13).

The lesion locations were as follows: upper thoracic esophagus (four lesions [31%]), middle thoracic esophagus (eight lesions [61%]), and lower thoracic esophagus (one lesion [8%]). All lesions involved less than one‐quarter of the esophageal circumference. Macroscopic type was classified as 0‐IIb in three lesions (23%) and 0‐IIc in 10 lesions (77%). The median endoscopic tumor size was 5 mm (range, 2–15 mm).

### 3.2 Therapeutic Outcomes of GI‐EMR With Submucosal Injection

GI‐EMR with submucosal injection was attempted for all eligible lesions, including those with post‐treatment scarring. In one lesion located on a post‐chemoradiotherapy (post‐CRT) scar, adequate lifting could not be achieved despite submucosal injection, and safe snaring was considered difficult due to severe fibrosis; therefore, the procedure was discontinued. The lesion was subsequently treated with ESD and was successfully resected. The technical success rate was 92% (12/13). Resection outcomes were evaluated on an intention‐to‐treat basis (*n* = 13) (Table 3). The endoscopic and histological en bloc resection rates were both 85% (11/13). Among the 12 successfully resected lesions, the only piecemeal resection occurred in a lesion on a post‐ESD scar, which was resected in two pieces, resulting in an Rx margin status. The median procedure time was 4.25 min (range, 2.0–11.0 min). The median specimen size was 9.5 mm (range, 3–20 mm), and the median maximum thickness of the submucosal layer included in the specimen was 403.5 µm (range, 161–735 µm). Histopathologically, five lesions (42%) were classified as intraepithelial neoplasia, while seven (58%) were diagnosed as squamous cell carcinoma; among the latter, six were confined to the EP and one to the LPM.

**TABLE 3:** Treatment outcomes and safety of gel immersion endoscopic mucosal resection (GI‐EMR) with submucosal injection.

Variables	Thirteen attempted lesions/12 resected lesions/nine patients
**Therapeutic outcomes**	
Histological en bloc resection rate, *n* (%)	11/13 (85%)
Technical success rate, *n* (%)	12/13 (92%)
Endoscopic en bloc resection rate, *n* (%)	11/13 (85%)
Procedure time, median (range), min	4.25 (2.0–11.0)
Specimen size, median (range), mm	9.5 (3–20)
Maximum thickness of submucosal tissue included in the resected specimen, median (range), µm	403.5 (161–735)
**Safety and adverse events**	
Intraoperative/delayed perforation, *n*	0/0
Post‐procedural bleeding, *n* (%)	0 (0%)
Gel‐related adverse events, *n* (%)	0 (0%)
**Post‐procedural invasiveness**	
Fever (>38°C) on post‐procedural day 1, *n* (%)	0 (0%)
White blood cell count on post‐procedural day 1, median (range), /µL	7795 (4820–13,050)
C‐reactive protein on post‐procedural day 1, median (range), mg/dL	0.28 (0.15–1.93)
**Histopathological findings**	
Intraepithelial neoplasia, *n* (%)	5 (42%)
EP carcinoma, *n* (%)	6 (50%)
LPM carcinoma, *n* (%)	1 (8%)

Technical success and endoscopic and histological en bloc resection rates were analyzed per attempted lesion (*n* = 13). Procedure time, specimen size, maximum submucosal thickness, and histopathological findings were analyzed per successfully resected lesion (*n* = 12). Post‐procedural fever, white blood cell count, and C‐reactive protein were analyzed per patient (*n* = 9).

### 3.3 Safety and Post‐Procedural Invasiveness

No cases of intraoperative or delayed perforation, post‐procedure bleeding, or gel‐related adverse events were observed. None of the patients developed a fever >38°C on the day after the procedure. The median white blood cell count was 7795/µL (range, 4820–13,050/µL), and the median C‐reactive protein level was 0.28 mg/dL (range, 0.15–1.93 mg/dL), indicating no significant inflammatory response. All patients followed an uneventful post‐procedure course and were discharged according to the planned postoperative management protocol.

## 4 Discussion

To the best of our knowledge, this is the first clinical case series evaluating GI‐EMR with submucosal injection for superficial esophageal squamous lesions. The technique evaluated in this study differs from conventional GI‐EMR in that submucosal injection was intentionally performed before snaring. In this study, we demonstrated the technical feasibility of GI‐EMR with submucosal injection for selected small superficial esophageal squamous lesions, with no procedure‐related adverse events observed. This technique achieved high endoscopic and histological en bloc resection rates. Furthermore, the procedure time was notably short, reflecting the simplicity of the technique and the stable visualization provided by GI. Therefore, GI‐EMR with submucosal injection may serve as a rapid, visually controlled resection strategy in carefully selected cases.

The procedure time observed in this study was markedly shorter than that reported in prior studies of conventional esophageal ESD, where the median or mean procedure times were approximately 40–85 min [16, 17, 18]. This substantial reduction in procedure time translates into a decreased procedural burden for both patients and endoscopists. Furthermore, it reduces the duration of sedation, which has been associated with adverse respiratory events, such as aspiration and pneumonia during prolonged upper gastrointestinal endoscopic procedures. Therefore, GI‐EMR with submucosal injection may be particularly advantageous in patients at higher risk of sedation‐related complications.

The favorable outcomes observed in this study may be attributable to the physical properties of the gel. In the narrow lumen of the esophagus, the stable capture of flat lesions can be challenging with conventional EMR [19]. Under immersion conditions, buoyancy may facilitate easier grasping of the lesion [20]. The gel is also more easily retained within a narrow lumen and suppresses the dispersion of blood and luminal contents, thereby stabilizing the visual field [21]. Furthermore, a heat‐sink effect has been suggested in colorectal GI‐EMR, which may help localize thermal injury during electrocautery and reduce any cautery‐related damage at the specimen margins [11]. A similar mechanism may have contributed to the favorable resection outcomes observed in this study. Although immersion of the lesion in gel may raise concern regarding visualization of the snare margins, the transparency of the gel allowed direct visualization of the lesion markings and snare during lesion capture in this series. Gel immersion also allowed maintenance of the working space without gas insufflation and helped stabilize the visual field by limiting the dispersion of blood and luminal contents. These characteristics may potentially facilitate controlled snaring while avoiding excessive luminal distension. However, because this study did not include a comparator group undergoing conventional EMR under gas insufflation, these potential advantages should not be interpreted as evidence of superiority over conventional EMR.

Procedural difficulty may also be influenced by the circumferential location of the lesion because gravity affects fluid accumulation, visualization, and tissue manipulation during esophageal endoscopic resection. In particular, gravity‐dependent lesions may be technically challenging. Gel immersion may potentially reduce this influence by maintaining a stable immersion environment around the lesion irrespective of gravity [12]. However, in the present study, we did not systematically evaluate procedural difficulty according to circumferential lesion location, and the small number of lesions precludes any definitive conclusion regarding location‐dependent differences in technical ease.

GI‐EMR with submucosal injection should not be considered a replacement for ESD, which remains the standard treatment for achieving reliable en bloc resection in larger or more complex lesions. Nevertheless, it represents a practical alternative to conventional EMR techniques in selected small superficial lesions. Conventional EMR methods, such as EMR‐C and ligation‐based resection, are typically performed without any direct visualization of the lesion during snaring and require dedicated devices [6, 7]. A technique such as two‐channel EMR is technically demanding, requires specialized equipment, and achieves only variable en bloc resection rates [14]. By contrast, GI‐EMR with submucosal injection allows direct visualization during snaring without the need for any additional devices, thereby facilitating more controlled and simplified resection. In the present study, all lesions were small (≤15 mm) and confined to the mucosa, representing favorable candidates for endoscopic resection. The applicability of this technique to larger lesions or those with significant submucosal fibrosis currently remains uncertain.

The thickness of submucosal tissue contained in an EMR specimen provides a histological measure of the depth of tissue captured during resection. Previous studies of multiband mucosectomy and EMR‐C reported median maximum submucosal thicknesses of approximately 750–900 µm [6, 7]. In the present study, the median maximum submucosal thickness was 403.5 µm. Although this value was smaller than those reported for suction‐based EMR techniques, the resected specimens contained submucosal tissue, allowing histopathological assessment of tumor invasion depth and the vertical resection margin. This difference may partly reflect the different resection mechanics of direct snaring under GI without suctioning the lesion into a cap or ligation device. However, the clinical significance of differences in submucosal thickness among EMR techniques remains unclear. Therefore, in the present study, submucosal thickness should primarily be interpreted as a histological indicator characterizing the depth of tissue captured by this technique.

Although the ability to perform resection without submucosal injection is an important advantage of underwater EMR and conventional GI‐EMR, submucosal injection was incorporated into the present technique based on our initial experience. We initially attempted snaring under GI without submucosal injection; however, adequate capture of the lesion was difficult because sufficient protrusion and mobility of the lesion could not be obtained. Submucosal injection was therefore added to create a submucosal cushion and facilitate controlled and reliable snaring. Considering the thin wall of the esophagus, the submucosal cushion was also considered to provide an additional safety margin during resection [19, 22, 23]. Whether submucosal injection is necessary for GI‐EMR in the esophagus remains to be determined, and conventional GI‐EMR without submucosal injection may be feasible in selected lesions.

One potential advantage of gel over water is its ability to remain at the target site because of its viscoelastic properties, allowing a stable immersion environment to be maintained with a smaller volume of fluid. In a previous study directly comparing GI‐EMR with underwater EMR for duodenal lesions, the amount of gel required for adequate immersion was significantly smaller than the amount of water required for underwater EMR [10]. Although this comparison was performed in duodenal lesions, this property may be particularly relevant in the esophagus, where proximal reflux of instilled fluid into the pharynx and subsequent aspiration are concerns during immersion‐based procedures. Indeed, head‐up positioning has been used during esophageal underwater EMR to reduce the risk of aspiration [24]. Thus, GI may offer a practical advantage over underwater EMR by maintaining a stable immersion environment with a smaller volume of instilled fluid. However, because the present study did not directly compare these techniques, this potential advantage requires further evaluation in comparative studies.

GI‐EMR with submucosal injection may also have potential clinical value in patients requiring repeated treatments. Superficial esophageal squamous neoplasms are known to arise metachronously and multifocally in the context of field cancerization [25]. Therefore, the availability of a minimally invasive technique that can be performed in a short time may be advantageous in such patients. However, the optimal role of GI‐EMR with submucosal injection in this setting requires further investigation. Conversely, the indications for GI‐EMR with submucosal injection may be limited in the presence of fibrosis. In the present study, the only lesion that required piecemeal resection was located on a post‐ESD scar, indicating that local fibrosis and reduced tissue mobility may hinder en bloc resection. In a lesion located on a post‐CRT scar, adequate lifting could not be obtained despite submucosal injection, and the procedure was discontinued because safe snaring was considered difficult in the presence of severe fibrosis. Radiation‐induced submucosal fibrosis may have reduced lesion elevation and mobility [26, 27]. Because snaring was not attempted after inadequate lifting was recognized, it remains uncertain whether resection might still have been feasible under GI despite insufficient elevation. Underwater EMR has been reported to be useful for fibrotic or non‐lifting lesions without submucosal injection in other gastrointestinal organs, and its feasibility has also been reported in the esophagus [28, 29]. These findings suggest that immersion‐assisted resection may remain feasible even when adequate lifting cannot be achieved by submucosal injection. Conventional GI‐EMR, which does not require submucosal injection, may also represent a potential option in such situations. Further studies are required to determine the optimal role of immersion‐based approaches for fibrotic or non‐lifting esophageal lesions.

This study has some limitations. First, it was a single‐center, retrospective case series with a small sample size, limiting the generalizability of the findings. Second, this study did not include a comparator group; therefore, no conclusions can be drawn regarding the superiority of GI‐EMR with submucosal injection over conventional EMR or ESD. All lesions were also small and limited to superficial mucosal disease, implying that the applicability of this technique to larger or fibrotic lesions remains uncertain. Finally, this study focused on short‐term outcomes, i.e., long‐term prognosis, recurrence rates, and cost‐effectiveness were not evaluated.

## 5 Conclusion

GI‐EMR with submucosal injection was technically feasible for selected superficial esophageal squamous lesions ≤15 mm. These findings suggest that this technique may represent a treatment option for appropriately selected small, non‐fibrotic mucosal lesions. Further studies with larger cohorts and comparative designs are required to determine its safety, optimal indications, and clinical role.

## Funding

The authors have nothing to report.

## Ethics Statement

**Approval of the research protocol by an Institutional Review Board**: This study was approved by the Institutional Review Board of Hiroshima University Hospital (approval number: E2025‐0296) and was conducted in accordance with the principles of the Declaration of Helsinki.

## Consent

Informed consent was obtained using an opt‐out approach through in‐hospital notification.

## Conflicts of Interest

The authors declare no conflicts of interest.

## Supporting information




**VIDEO S1**: Gel immersion endoscopic mucosal resection with submucosal injection for a superficial esophageal squamous lesion. After lesion marking, VISCOCLEAR gel was injected to immerse the lesion, followed by a submucosal injection of MucoUp, snaring under gel immersion, confirmation of the post‐resection ulcer base, and presentation of the pinned resected specimen showing the preoperative marking dots.

## Data Availability

The data that support the findings of this study are not publicly available because they contain clinical information that could compromise the privacy of research participants. The data are available from the corresponding author upon reasonable request and in accordance with institutional ethics regulations.
